# Reallocation of Soluble Sugars and IAA Regulation in Association with Enhanced Stolon Growth by Elevated CO_2_ in Creeping Bentgrass

**DOI:** 10.3390/plants11111500

**Published:** 2022-06-02

**Authors:** Jingjin Yu, Meng Li, Qiuguo Li, Ruying Wang, Ruonan Li, Zhimin Yang

**Affiliations:** 1 College of Agro-Grassland Science, Nanjing Agricultural University, Nanjing 210095, China; nauyjj@njau.edu.cn (J.Y.); 2018120014@stu.njau.edu.cn (M.L.); 2020820040@stu.njau.edu.cn (Q.L.); 2021120015@stu.njau.edu.cn (R.L.); 2 Department of Horticulture, Oregon State University, 4017 Agriculture and Life Sciences Building, Corvallis, OR 97331, USA; ruying.wang@oregonstate.edu

**Keywords:** elevated CO_2_, stolon growth, soluble sugars, hormone, creeping bentgrass

## Abstract

Extensive stolon development and growth are superior traits for rapid establishment as well as post-stress regeneration in stoloniferous grass species. Despite the importance of those stoloniferous traits, the regulation mechanisms of stolon growth and development are largely unknown. The objectives of this research were to elucidate the effects of the reallocation of soluble sugars for energy reserves and endogenous hormone levels for cell differentiation and regeneration in regulating stolon growth of a perennial turfgrass species, creeping bentgrass (*Agrostis stolonifera* L.). Plants were grown in growth chambers with two CO_2_ concentrations: ambient CO_2_ concentration (400 ± 10 µmol mol^−1^) and elevated CO_2_ concentration (800 ± 10 µmol mol^−1^). Elevated CO_2_ enhanced stolon growth through increasing stolon internode number and internode length in creeping bentgrass, as manifested by the longer total stolon length and greater shoot biomass. The content of glucose, sucrose, and fructose as well as endogenous IAA were accumulated in stolon nodes and internodes but not in leaves or roots under elevated CO_2_ concentration. These results illustrated that the production and reallocation of soluble sugars to stolons as well as the increased level of IAA in stolon nodes and internodes could contribute to the enhancement of stolon growth under elevated CO_2_ in creeping bentgrass.

## 1. Introduction

Stolon is an elongated axillary shoot composed of nodes, internodes, and leaves [1,2]. Stolon nodes contain meristematic tissues which are capable of producing adventitious roots and offspring ramets from nodes [3,4]. The benefits of clonality include rapid local spread through stolon growth as well as a high stand establishment rate due to the physiological connections between ramets to share resources of carbohydrates, nutrients and water [3,5,6]. Stoloniferous plants can be clonally propagated and have advantageous traits such as rapid establishment and recovery from stresses. In turfgrass, vegetative propagation (stolon cuttings) or sprigging is a commonly and extensively used method for rapid turf production and establishment utilizing stolon cuttings in warm-season grass species [7,8,9]. Therefore, rapid stolon growth is one of the highly desirable characteristics of stoloniferous turfgrass species.

The atmospheric CO_2_ concentration has risen from the pre-industrialized era 280 µmol mol^−1^ to the present 400 µmol mol^−1^ and will reach over 1000 µmol mol^−1^ by the end of this century according to IPCC [10,11]. Therefore, research interests in plant responses to elevated CO_2_ are increasing [12]. A large number of studies reported that elevated CO_2_ could promote plant growth and development, including perennial grass species, such as tall fescue (*Festuca arundinacea* Schreb.) [13,14,15,16], Kentucky bluegrass (*Poa pratensis* L.) [17,18], and bermudagrass (*Cynodon dactylo*n (L.) Pers.) [19,20]. However, few previous studies about elevated CO_2_-induced effects were found focusing on stolon growth. 

Many studies documented that elevated CO_2_-induced promotion in plant growth was associated with changes in carbohydrates content via stimulating photosynthetic capacity for synthesizing carbohydrates in plants [18,20,21]. For example, elevated CO_2_ led to a significant increase in total non-structural carbohydrates in the leaves of Kentucky bluegrass [17]. Kinmonth-Schultz and Kim [22] found that elevated CO_2_ improved fructan accumulation in the underground rhizomes in order to overwinter and spread in reed canary grass (*Phalaris arundinacea* L.). In stolon tips of creeping bentgrass (*Agrostis stolonifera*), there was a significant decrease in fructose and sucrose as well as an increase in maltose under elevated CO_2_ conditions through GC-MS analysis [2]. Burgess and Huang [21] found that elevated CO_2_ caused an increase in the total stolon length as well as net photosynthetic rate in creeping bentgrass. Such an increase in total stolon length was attributed to the increased stolon internode number [2]. 

Apart from carbohydrates, plant hormones are also of great importance in affecting growth and development via biosynthesis, degradation, transport, and signaling to regulate multiple biological processes in plants [23]. Among several common endogenous hormones, auxins (including indole-3-acetic acid; IAA), cytokinins (including isopentenyl adenosine; iPA), and gibberellic acids (GAs) are the most well-known ones in controlling cell division and elongation during plant growth and development due to their regulatory roles in each biological process from embryogenesis to maturity in various plant species [24,25,26]. In potato (*Solanum tuberosum* L.), IAA and GA_3_ were found to be essential for stolon elongation [25]. In creeping bentgrass, Burgess et al. [27] reported that elevated CO_2_ did not alter endogenous iPA or IAA in the leaves under well-watered conditions but increased the content of iPA and decreased IAA under drought stress. The GA regulation under elevated CO_2_ is still unknown because the contents of GAs were not measured in that study [27]. As illustrated in our previous study, elevated CO_2_ caused increases in total stolon length by some metabolites involved in carbohydrate reserves, respiratory metabolism, and membrane maintenance in the stolon tips of creeping bentgrass [2]. Nevertheless, very limited knowledge is currently available about the effects of elevated CO_2_ on stolon growth with respect to soluble sugars and endogenous hormones allocation in different perennial plant organs such as root, leaf, node, and internode in stoloniferous grass species. 

We hypothesized that elevated CO_2_ improvement on stolon growth might be associated with the reallocation of soluble sugars for energy reserves and endogenous hormone levels for cell differentiation and regeneration in regulating stolon growth of a perennial turfgrass species, creeping bentgrass. Understanding the specific soluble sugars and hormones in different organs in response to elevated CO_2_ concentration will provide some new insights into mechanisms about how elevated CO_2_ enhances stolon growth of stoloniferous plants under the scenario of climate change in the future.

## 2. Results

### 2.1. Effects of Elevated CO_2_ on Morphological Parameters in Creeping Bentgrass

**Figure 1:**
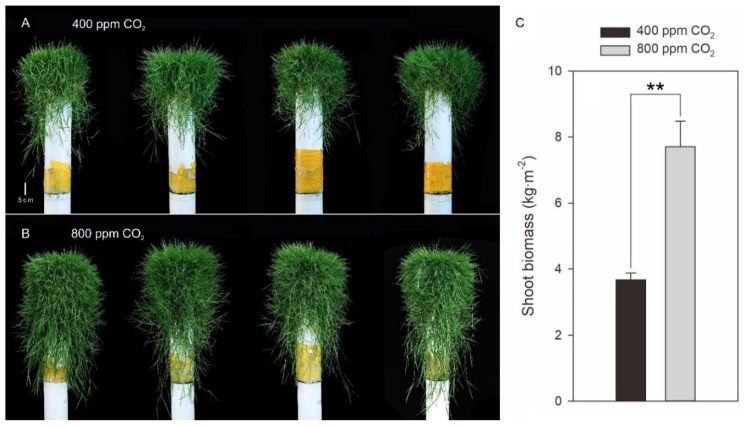
Effects of elevated CO_2_ concentration on shoot phenotype (**A**,**B**) and shoot biomass (**C**) of creeping bentgrass at 42 d of experiment. Four hundred µmol mol^−1^ (ppm) CO_2_, ambient CO_2_ concentration; 800 ppm CO_2_, elevated CO_2_ concentration. ** indicates a significant difference between ambient and elevated CO_2_ concentrations according to Student’s *t*-test at *p* ≤ 0.01. Error bars represent standard error (SE).

The phenotypic responses of creeping bentgrass to CO_2_ levels were dramatically different as shown in Figure 1A,B, indicating that elevated CO_2_ significantly enhanced stolon growth compared to ambient CO_2_. Shoot biomass was positively correlated with total stolon length, internode length, internode number, and root biomass (Table 1) and significantly increased by 1.10-fold due to elevated CO_2_ (Figure 1C). 

**Figure 2:**
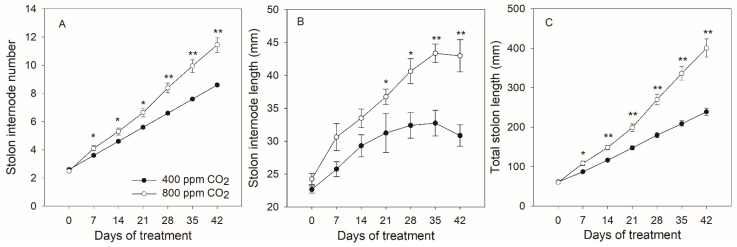
Effects of elevated CO_2_ concentration on stolon internode number (**A**), stolon internode length (**B**), and total stolon length (**C**) of creeping bentgrass at 42 d of experiment. Four hundred µmol mol^−1^ (ppm) CO_2_, ambient CO_2_ concentration; 800 ppm CO_2_, elevated CO_2_ concentration. * and ** indicate a significant difference between ambient and elevated CO_2_ concentrations according to Student’s *t*-test at *p* ≤ 0.05 and *p* ≤ 0.01, respectively. Error bars represent standard error (SE).

Total stolon length was positively correlated with stolon internode length, internode number, and shoot and root biomass (Table 1). Elevated CO_2_ significantly enhanced stolon internode number and total stolon length from 7 to 42 d, and stolon internode length from 21 to 42 d of the experimental period (Figure 2). The stolon internode number of creeping bentgrass grown under elevated CO_2_ was consistently greater than under ambient CO_2_ and the differences in internode were increased from an average of 0.5 at 7 d to 2.9 at 42 d (Figure 2A). At the conclusion of the study, the elevated CO_2_-caused increase in stolon internode length reached 12.1 mm at 42 d in comparison with the ambient CO_2_ concentration (Figure 2B). 

**Figure 3:**
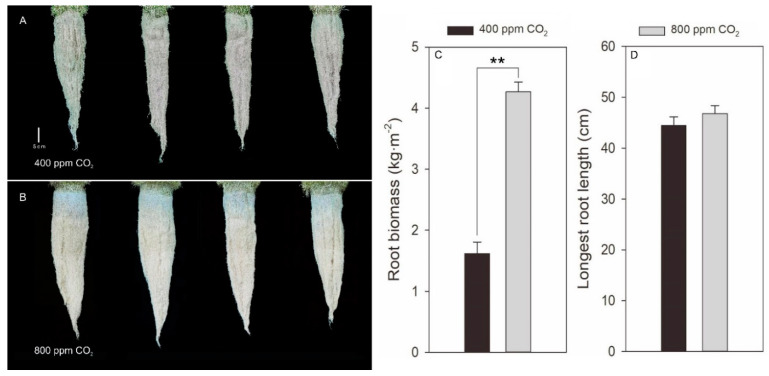
Effects of elevated CO_2_ concentration on root phenotype (**A**,**B**), root biomass (**C**), and longest root length (**D**) of creeping bentgrass at 42 d of experiment. Four hundred µmol mol^−1^ (ppm) CO_2_, ambient CO_2_ concentration; 800 ppm CO_2_, elevated CO_2_ concentration. ** indicates a significant difference between ambient and elevated CO_2_ concentrations according to Student’s *t*-test at *p* ≤ 0.01. Error bars represent standard error (SE).

In addition to proliferated shoot growth, elevated CO_2_ also stimulated substantial root growth of creeping bentgrass (Figure 3A,B). Root biomass was positively correlated with total stolon length, internode length, internode number, and shoot biomass (Table 1). Elevated CO_2_ significantly increased root biomass by 1.64-fold at 42 d of experiment in comparison with ambient CO_2_ (Figure 3C). However, no difference was found in the longest root length of creeping bentgrass between elevated and ambient CO_2_ concentrations (Figure 3D).

### 2.2. Effects of Elevated CO_2_ on Shoot Soluble Sugars

**Figure 4:**
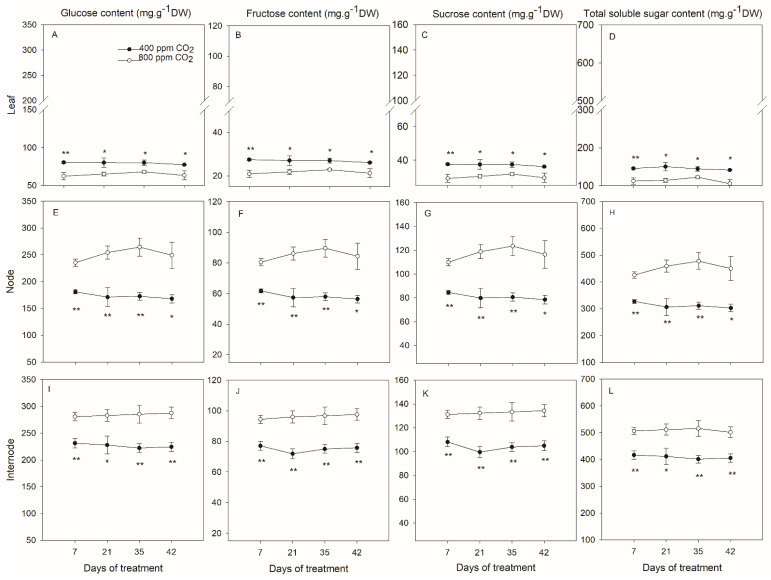
Effects of elevated CO_2_ concentration on content of glucose (**A**,**E**,**I**), fructose (**B**,**F**,**J**), sucrose (**C**,**G**,**K**) and total soluble sugar (**D**,**H**,**L**) in the leaf (**A**–**D**), node (**E**–**H**), and internode (**I**–**L**) tissues of creeping bentgrass at 42 d of experiment. Four hundred µmol mol^−1^ (ppm) CO_2_, ambient CO_2_ concentration; 800 ppm CO_2_, elevated CO_2_ concentration. Sugar contents are presented in the unit of mg g^−1^ dry weight (DW). * and ** indicate a significant difference between ambient and elevated CO_2_ concentrations according to Student’s *t*-test at *p* ≤ 0.05 and *p* ≤ 0.01, respectively. Error bars represent standard error (SE).

In creeping bentgrass, soluble sugar levels were generally lowered in the leaf tissue than in the node and internode (Figure 4). In the leaves, elevated CO_2_ caused significant decreases in all soluble sugars measured in this study from 7 to 42 d of experiment (Figure 4A–D). More specifically, glucose content in the leaves in response to elevated CO_2_ was reduced by 29.0%, 32.6%, 17.8%, and 33.9% at 7, 21, 35, and 42 d, respectively, in comparison with ambient CO_2_ (Figure 4A); and reduction in fructose content was 31.5%, 30.6%, 18.3%, and 35.0% (Figure 4B) and in sucrose content was 29.1%, 32.6%, 17.8%, and 33.9% (Figure 4C) at 7, 21, 35, and 42 d, respectively. Therefore, the content of total soluble sugars was significantly decreased by 22.8%, 24.1%, 15.2%, and 25.4% at 7, 21, 35, and 42 d, respectively, under elevated CO_2_ concentration (Figure 4D).

Three soluble sugars and total soluble sugars exhibited greater accumulation in the stolon nodes in response to elevated CO_2_, which was the opposite change observed in the leaves of creeping bentgrass (Figure 4). In the stolon nodes, glucose content under elevated CO_2_ was 30.0%, 48.7%, 53.2%, and 48.4% higher (Figure 4E) and fructose content was 30.4%, 50.1%, 54.6%, and 49.8% higher (Figure 4F) than that under ambient CO_2_ at 7, 21, 35, and 42 d, respectively. Similarly, elevated CO_2_ resulted in a significant increase in sucrose content from 7 to 42 d of treatment time in comparison with ambient CO_2_ (Figure 4G). Hence, total soluble sugar in the stolon nodes of creeping bentgrass was significantly enhanced by 30.1%, 49.8%, 53.5%, and 48.7% at 7, 21, 35, and 42 d, respectively, under elevated CO_2_ concentration (Figure 4H). 

Similar to the responses observed in the stolons, soluble sugars including glucose, fructose, and sucrose as well as total soluble sugar contents also increased under elevated CO_2_ compared with ambient CO_2_ in the stolon internodes of creeping bentgrass (Figure 4). Elevated CO_2_ significantly increased the glucose content by 21.4%, 24.2%, 28.4%, and 28.1% (Figure 4I), and fructose content by 22.5%, 33.5%, 29.0%, and 28.6% at 7, 21, 35, and 42 d of treatments, respectively (Figure 4J). The sucrose content was significantly enhanced by elevated CO_2_ in consistence with glucose and fructose at 7, 21, 35, and 42 d of treatments (Figure 4K). Therefore, total soluble sugars in the internodes increased by 21.6%, 24.3%, 28.5%, and 28.2% at 7, 21, 35, and 42 d, respectively, due to elevated CO_2_ compared with ambient CO_2_ concentration (Figure 4L). 

### 2.3. Effects of Elevated CO_2_ on Root Soluble Sugars

**Figure 5:**
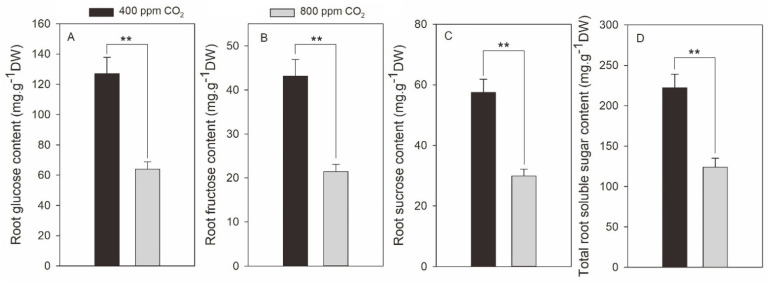
Effects of elevated CO_2_ concentration on root content of glucose (**A**), fructose (**B**), sucrose (**C**) and total soluble sugar (**D**) in creeping bentgrass at 42 d of experiment. Four hundred µmol mol^−1^ (ppm) CO_2_, ambient CO_2_ concentration; 800 ppm CO_2_, elevated CO_2_ concentration. Sugar contents are presented in the unit of mg g^−1^ dry weight (DW). ** indicates a significant difference between ambient and elevated CO_2_ concentrations according to Student’s *t*-test at *p* ≤ 0.01. Error bars represent standard error (SE).

Soluble sugars, including glucose, fructose, sucrose, and total soluble sugars, in the roots, decreased when plants were exposed to elevated CO_2_ (Figure 5). In response to elevated CO_2_, root glucose and fructose contents significantly declined by 49.7% and 50.4%, respectively (Figure 5A,B). Elevated CO_2_ also resulted in a reduction in root sucrose content by 48.0% in comparison with ambient CO_2_ (Figure 5C). Collectively, the total soluble sugar contents in the roots were significantly decreased by 49.8% under elevated CO_2_ compared with ambient CO_2_ concentration (Figure 5D).

### 2.4. Effects of Elevated CO_2_ on Endogenous Hormone Content

**Figure 6:**
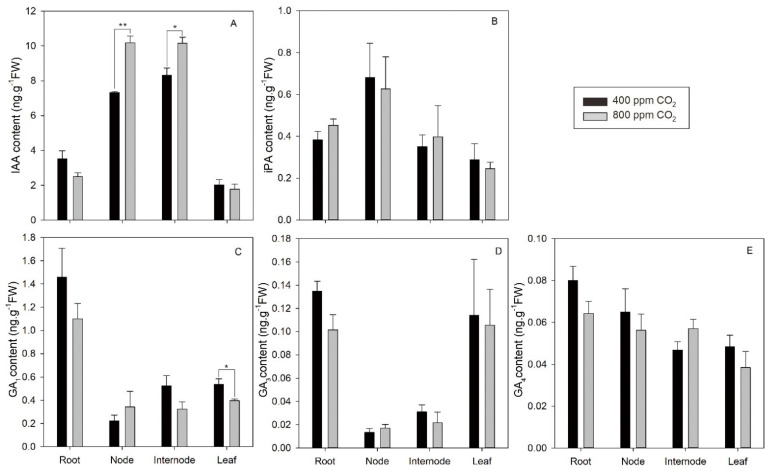
Effects of elevated CO_2_ concentration on content of IAA (**A**), iPA (**B**), GA_1_ (**C**), GA_3_ (**D**), and GA_4_ (**E**) in the root, node, internode, and leaf tissues of creeping bentgrass at 42 d of experiment. Hormone contents are presented in the unit of ng g^−1^ fresh weight (FW). Four hundred µmol mol^−1^ (ppm) CO_2_, ambient CO_2_ concentration; 800 ppm CO_2_, elevated CO_2_ concentration. * and ** indicate a significant difference between ambient and elevated CO_2_ concentrations according to Student’s *t*-test at *p* ≤ 0.05 and *p* ≤ 0.01, respectively. Error bars represent standard error (SE).

Endogenous IAA, iPA, GA_1_, GA_3_, and GA_4_ levels in different plant tissues including root, node, internode, and leaf are shown in Figure 6. Compared with ambient CO_2_, the content of IAA was significantly increased by elevated CO_2_ in both nodes and internodes by 39.0% and 22.1%, respectively, but not in roots or leaves (Figure 6A). No difference was found in the contents of iPA, GA_3_, or GA_4_ in plants grown under elevated CO_2_ (Figure 6B,D,E). Among all the tissues tested, only leaf exhibited a significant decrease in GA_1_ content due to elevated CO_2_ in comparison with ambient CO_2_ (Figure 6C).

## 3. Discussion

Extensive stolon development and growth are superior traits for the rapid establishment as well as post-stress regeneration for survival in stoloniferous grass species. Although stolon initiation and formation are mainly controlled by genetic factors, the growth and development of stolon are often influenced by diverse factors. Previous reports have demonstrated that changes in stolon internode length and rhizome length were associated with several factors such as temperature, nitrogen application, water availability, stolon internode position, burial depth of stolon internode as well as elevated CO_2_ [28,29,30,31,32,33]. For example, the total rhizome length of Kentucky bluegrass exposed to drought stress was significantly lower under ambient CO_2_ but unchanged under elevated CO_2_ concentration [33]. In our study, elevated CO_2_ stimulated the aboveground stolon growth through increases in stolon internode number and length, these morphological changes lead to more than double the shoot biomass of creeping bentgrass grown under ambient CO_2_ levels (Figure 1 and Figure 2). In other stoloniferous plants, increased stolon internode length as well as stolon thickness could enhance the survival rate and regeneration capacity due to the increased amount of reserves such as soluble proteins, starch, and soluble sugars in the stolons [34,35,36]. In this study, longer stolon internode length suggested the tendency of creeping bentgrass to spread horizontally to sustain the enhanced photosynthetic capacity under elevated CO_2_. The greater shoot biomass was a result of the dramatic stolon elongation as well as the increase in leaf number (data not shown) which was due to the increased stolon internode number. The potential mechanisms of CO_2_-induced stolon elongation involved in metabolic pathways in stoloniferous creeping bentgrass are discussed below, including soluble sugars (glucose, fructose, and sucrose), endogenous hormones (IAA, iPA, and GAs), and root growth and development. 

Elevated CO_2_-enhanced plant growth is a common response and has been well documented in various plant species without stolons [13,17,20]. In this study, the proliferative shoot growth under elevated CO_2_ was in fact a result of the significant increase in stolon growth (Figure 1 and Figure 2). Interestingly, the contents of soluble sugars glucose, fructose, and sucrose were increased significantly in stolon nodes and internodes but decreased in leaves and roots in creeping bentgrass subjected to elevated CO_2_ concentration (Figure 4 and Figure 5), suggesting that soluble carbohydrates were allocated to stolons for storage rather than to leaves and roots in a stoloniferous plant. In other plants without stolons, elevated CO_2_ did not lead to a decline in soluble sugars in leaves such as radish (*Raphanus sativus* L.) [37], barley (*Hordeum vulgare* L.) cultivars [38], cork oak (*Quercus suber* L.) [39], and perennial Kentucky bluegrass [17,18]. Therefore, the results of soluble sugars revealed that the positive effects of elevated CO_2_ in stolon growth are likely to be attributed to the increased photosynthetic carbon acquisitor as well as the alteration in carbon reallocation [40].

Resources including soluble carbohydrates, starch, mineral nutrients, and soluble protein stored in stolon nodes and internodes may be responsible for plant survival and regeneration in order to cope with severe disturbance when plants are disturbed by various biotic and abiotic factors [4,41,42,43]. The content of carbohydrates in stolon nodes and internodes was positively correlated with the survival rate of stoloniferous plants [44]. In order to quickly establish in the soil, zoysiagrass (*Zoysia* spp.) genotypes with greater total stolon length were demonstrated to distribute more dry matter to stolons and rhizomes instead of leaves [45]. In this study, the soluble sugars from leaves and roots were reallocated to stolon nodes and internodes to support the enhanced stolon growth under elevated CO_2_ concentration. The increased stolon growth and carbohydrates storage may explain the mechanism behind the elevated CO_2_ enhanced survival and recovery traits under abiotic stresses such as heat [19], drought [27,46], and salinity [20] in grass species with stolons.

It is interesting to find that root biomass under elevated CO_2_ was significantly higher than that under ambient CO_2_ conditions, although the content of soluble sugars in root was decreased by elevated CO_2_ in this study (Figure 3 and Figure 5). The increase in root biomass was attributed to the greater root density but not the root length as indicated by root phenotype under elevated CO_2_ (Figure 3). Our observation of root biomass was in accordance with other studies which were also conducted in creeping bentgrass [21,47]. In other plants, the improvement of elevated CO_2_ on root density was mainly due to elevated CO_2_-induced formation and development of lateral roots and fine roots as reported in *Sedum alfredii* Hance. [48] and maize (*Zea mays* L.) [49]. The lower root soluble sugars content was likely due to the consumption for producing greater root biomass in combination with the reallocation of soluble sugars from roots to stolon nodes and internodes under elevated CO_2_ conditions. Our study is the first report that examined the stimulation of elevated CO_2_ on stolon growth from carbon reallocation among root, leaf, node, and internode tissues in stoloniferous plant species.

Hormones are crucial regulators of plant growth and development; hence, plants might alter their hormone levels to regulate plant growth in response to elevated CO_2_ conditions. Early research has demonstrated that IAA plays important roles in regulating stolon growth and development by cell division and cell elongation [50]. Exogenous IAA applied at the distal end of decapitated stolons in *Saxifraga sarmentosa* L. enhanced the translocation of ^14^C assimilates from the leaf into the stolon [50]. Exogenous cytokinin increased auxin content in the stolon tips of potato resulting in tuber initiation [22]. In our study, elevated CO_2_ increased the endogenous level of IAA in both stolon nodes and internodes but not in roots or leaves in creeping bentgrass (Figure 6). The result indicated that elevated CO_2_ not only directly promoted carbon fixation through photosynthesis but also regulated growth by controlling endogenous auxin levels. This would explain why the higher content of IAA in stolon nodes and internodes but not in leaves or roots was in consistence with the allocation of soluble sugars. The unchanged IAA level in response to elevated CO_2_ in leaf was also observed by Burgess et al. [27] in creeping bentgrass under unstressed conditions. The accumulation of IAA in stolon node and internode implied that increased endogenous IAA content could have provided a great contribution to the rapid stolon elongation and growth in creeping bentgrass exposed to elevated CO_2_ conditions. 

Apart from auxins, cytokinins and GAs are generally believed to serve as positive regulators of plant growth and development [51,52]. In this study, no significant difference was found in the content of iPA and GAs (GA_1_, GA_3_, and GA_4_) in the stolons or roots of creeping bentgrass grown under ambient and elevated CO_2_ concentrations (Figure 6B–E). Similarly, exogenous kinetin and GA to the distal zone of stolons in *Saxifraga sarmentosa* generated a small insignificant effect in promoting stolon growth [50]. Auxin was reported to inflict a negative effect on cytokinins by inhibiting IPT expression and enhancing CYTOKININ OXIDASE/DEHYDROGENASE (CKX) expression to reduce the content of cytokinins in different species [53]. Therefore, the significant increase in IAA may have inhibited the production of iPA in stolon nodes and internodes under elevated CO_2_ conditions. Furthermore, we also observed a reduction in GA_1_ in leaves under elevated CO_2_ (Figure 6C). Adjusting the GA_1_ concentration in plants has great practical uses. Plant growth regulators, such as trinexapac-ethyl, were utilized to inhibit GA_1_ production. In particular, trinexapac-ethyl blocks the conversion of metabolically inactive GA_20_ to active GA_1_ [54]. Trinexapac-ethyl is one of the most widely used plant growth regulators in turfgrass management and numerous research reports have demonstrated its benefits to turfgrass with improved tolerance to biotic and abiotic stresses [55]. In creeping bentgrass, trinexapac-ethyl improved drought and heat tolerance [56,57]. The reduction in GA_1_ in the leaves due to elevated CO_2_ could have contributed to increased tolerance to other stresses (such as heat and drought) in a similar way as regulated by trinexapac-ethyl. However, this speculation will require further investigation. Our study quantified endogenous hormones in different tissue types of creeping bentgrass and therefore provided important evidence suggesting that elevated CO_2_-induced stolon elongation resulted from IAA increase but not iPA or GAs in stolon nodes and internodes. In response to elevated CO_2_ concentration, the decreased GA_1_ level from this research also supported the shorter leaf length observed by Burgess and Huang [21] in the same species, creeping bentgrass. Therefore, the proposed hormone regulation model for creeping bentgrass was that elevated CO_2_ promoted lateral growth but not vertical growth by increasing the IAA level in stolons and decreasing the GA_1_ level in leaves.

## 4. Materials and Methods

### 4.1. Plant Material and Growth Conditions

Creeping bentgrass (cv. ‘Penn-A4′) stolons with the same number of nodes were planted in polyvinyl chloride (PVC) tubes (10 cm in diameter and 50 cm in depth) filled with sand. Plants were established for about three months from July to September 2020 in a greenhouse with an average temperature of 25/20 °C (day/night), PAR of 450 µmol m^−2^ · s^−1^, and 14 h photoperiod. Plants were trimmed twice a week to promote density and irrigated with Hoagland solution [58] once a week. After establishment, plants were acclimated in a growth chamber (Xubang, Jinan, China) with the temperature set at 25/20 °C (day/night), 70% relative humidity, PAR of 600 µmol m^−2^ · s^−1^ at the canopy level, and a 14 h photoperiod for one week before treatments initiation. 

### 4.2. Experimental Design and Treatments

The experiment was initiated on 23rd October in 2020 with five replications of two CO_2_ treatments: ambient CO_2_ concentration (400 ± 10 µmol mol^−1^) and elevated CO_2_ concentration (800 ± 10 µmol mol^−1^). The CO_2_ concentration of growth chambers was automatically controlled through an open-chamber control system via computer programs connected to a CO_2_ gas tank with 100% CO_2_ [2]. During the experiment, PVC pots were randomly relocated every other day within and across chambers to avoid spatial environmental variations in chambers.

### 4.3. Growth and Physiological Measurements

The impacts of elevated CO_2_ on stolon growth were evaluated by measuring stolon internode length and stolon internode number on each individual stolon as well as the total stolon length of plants in each pot according to Xu et al. [2] with minor modifications. Four individual stolons were labeled in each pot at 0 d of treatments. The internode length for each labeled stolon and longest root length were measured by a ruler. The internode numbers of each labeled stolon were counted on every sampling day. The total stolon length was measured from the labeled point to the tip of each stolon. 

Biomass of shoot and root was measured by drying the total tissues from each pot to a constant weight at 70 °C for 3 days at 42 d of experiment. The dry biomass weights were divided by the surface area of the PVC pot to report the sample biomass weight per unit area (kg m^−2^).

### 4.4. Sugar Extraction and Quantification

Soluble sugars including glucose, fructose, and sucrose were quantified using the phenol-sulfuric acid method described by Liu et al. [59] with modifications. Leaf samples at 7, 21, 35, and 42 days of treatment were collected and dried, then ground to a fine powder with a pestle. To extract soluble sugars, 25 mg of fine powder was mixed with 5.0 mL of 80% (*v*/*v*) aqueous ethyl alcohol in a 15 mL microcentrifuge tube and incubated in a water bath at 30 °C for 30 min. Microcentrifuge tubes were then centrifuged at 4500 rpm for 10 min to obtain supernatant. The supernatant was transferred to 50 mL microcentrifuge tubes and 2.5 mL of 80% (*v*/*v*) aqueous ethanol was added and extracted two times with the same method to obtain the final extractant. A subsample of 1 mL extractant was mixed with 1 mL 23% (*v*/*v*) phenol solution, then 5 mL 98% (*v*/*v*) concentrated sulfuric acid was added to the solution and mixed well. The reaction solution was cooled down to room temperature for 15 min and then incubated in a water bath at 30 °C for 30 min. The absorbance of the reaction solution at 490 nm was measured with a spectrophotometer (Ultrospec 2100 pro, Biochrom Ltd., Cambridge, UK). Glucose, fructose, and sucrose contents were quantified by comparing their standard curves. The total soluble sugars reported in this study were calculated as the sum of glucose, fructose, and sucrose.

### 4.5. Hormone Measurement

The extraction procedure of hormones (IAA, iPA, GA_1_, GA_3_, and GA_4_) was conducted according to the modified method by Pan et al. [60]. One gram of each leaf, node, internode, and root fresh sample was collected at 42 d from plants grown under different CO_2_ concentrations and ground to a fine powder in liquid nitrogen and then transferred into microcentrifuge tubes. A 10 mL isopropanol/hydrochloric extract buffer was added to tubes and shaken at 4 °C for 30 min, and 20 mL dichloromethane was added for an additional 30 min shaking at 4 °C. The solution was centrifuged at 4 °C, 12,000 rpm for 5 min, and the lower phase was concentrated by nitrogen evaporator into the dried precipitate which was dissolved in 200 μL methanol containing 0.1% formic acid. Then, the extraction was filtered by a 0.22 μm filter membrane for further hormones measurement.

Plant hormone samples were quantified using HPLC-MS/MS by 1290 HPLC (Agilent, Santa Clara, CA, USA) and SCIEX-6500 Qtrap (AB *Sciex*, Foster, CA, USA), following the parameters setup as described by Pan et al. [60]. Standards of plant hormones including IAA, iPA, GA_1_, GA_3_, and GA_4_ were ordered from Sigma-Aldrich and dissolved in methyl alcohol with 0.1% methanoic acid for the external standard curves. The HPLC conditions were: reverse-phase poroshell 120 SB-C^18^ chromatographic column (Agilent, Palo Alto, CA, USA) with a column temperature of 30 °C. Mobile phases A:B = (0.1% formic acid in methanol): (0.1% formic acid in water) was used for separation. The elution gradient was set as follows: 0–1 min A = 20%; 1–3 min A increased from 20% to 50%; 3–9 min A increased from 50% to 80%; 9–10.5 min A = −80%; 10.5–10.6 min A decreased from 80% to 20%; 10.6–13.5 min A = 20%. The injection volume was 2 μL. The MS conditions were set as follows: ionspray voltage 4500 v, source temperature 400 °C, curtain gas 15 psi, nebulizing gas 65 psi, auxiliary gas 70 psi.

### 4.6. Statistical Analyses

Data were analyzed using SPSS statistics software (SPSS 18.0; SPSS Inc., Chicago, IL, USA). The Pearson correlation analysis was used to analyze the effects of elevated CO_2_ on all parameters including shoot and root biomass, stolon internode number and length, total stolon length, and longest root length. The means ± standard error (SE) was summarized in charts for shoot biomass, root biomass, longest root length, internode number, internode length, total stolon length, shoot soluble sugar contents, root soluble sugar contents, and hormone contents. Student’s *t*-tests were used to determine significant differences at confidence levels of 0.05 and 0.01. 

## 5. Conclusions

**Figure 7:**
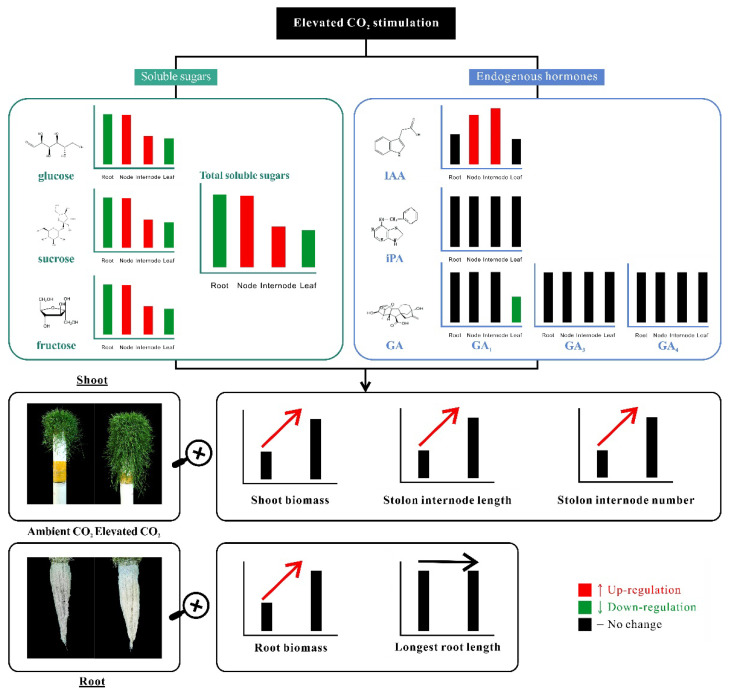
Working model for elevated CO_2_-responsive metabolic pathways associated with soluble sugars and endogenous hormones in regulating stolon growth in creeping bentgrass.

In conclusion, elevated CO_2_ enhanced the stolon growth by promoting stolon internode number, internode length, and root biomass in creeping bentgrass, as manifested by the longer total stolon length and greater shoot biomass. The regulatory model of the aforementioned carbohydrates and hormones which may be associated with stolon growth are summarized in Figure 7. The content of soluble sugars including glucose, sucrose, and fructose as well as endogenous IAA was accumulated in stolon nodes and internodes but not in leaves or roots under elevated CO_2_ concentration. These results illustrated that the accumulation and reallocation of glucose, sucrose, and fructose to stolons as well as the increased IAA level in stolon nodes and internodes could contribute to the enhancement of stolon growth under elevated CO_2_ in creeping bentgrass. Our study is an important step further in understanding the endogenous hormones and soluble sugars reallocation involved in elevated CO_2_-enhanced stolon growth. However, the molecular mechanism underlying the enhanced stolon development is still unknown. Research is needed to explore the detailed mechanisms as to how CO_2_-responsive soluble carbohydrates and IAA in stolon node and internode regulate stolon growth in creeping bentgrass in order to provide further insights into survival strategies by promoting stolon growth and biomass production of above-ground shoots.

## Figures and Tables

**Table 1:** Pearson Correlation analysis among growth parameters in creeping bentgrass.

	Total Stolon Length	Internode Length	Internode Number	Shoot Biomass	Root Biomass	Longest Root Length
Total stolon length	1	0.938 **	0.973 **	0.693 *	0.939 **	0.121
Internode length	0.938 **	1	0.883 **	0.686 *	0.831 **	0.121
Internode number	0.973 **	0.883 **	1	0.587	0.920 **	0.138
Shoot biomass	0.693 *	0.686 *	0.587	1	0.811 **	0.383
Root biomass	0.939 **	0.831 **	0.920 **	0.811 **	1	0.186
Longest root	0.121	0.121	0.138	0.383	0.186	1

Note: * and ** indicate significant correlation at 0.05 and 0.01 probability levels, respectively.

## Data Availability

Data are available upon request from the first author.

## References

[1] Savini G., Giorgi V., Scarano E., Neri D. (2008). Strawberry plant relationship through the stolon. Physiol. Plant..

[2] Xu Q., Fan N., Zhuang L., Yu J., Huang B. (2018). Enhanced stolon growth and metabolic adjustment in creeping bentgrass with elevated CO_2_ concentration. Environ. Exp. Bot..

[3] Pitelka L.E., Ashmun J.W., Jackson J.B.C. (1985). Physiology and integration of ramets in clonal plants. Population Biology and Evolution of Clonal Organisms.

[4] Stuefer J.F., Huber H. (1999). The role of stolon internodes for ramet survival after clone fragmentation in *Potentilla anserina*. Ecol. Lett..

[5] Fahrig L., Coffin D.P., Lauenroth W.K., Shugart H.H. (1994). The advantage of long-distance clonal spreading in highly disturbed habitats. Evol. Ecol..

[6] Song Y.B., Yu F.H., Li J.M., Keser L.H., Fischer M., Dong M., Kleunen M.V. (2013). Plant invasiveness is not linked to the capacity of regeneration from small fragments: An experimental test with 39 stoloniferous species. Biol. Invasions.

[7] Hanna W.W. (1995). Centipedegrass—Diversity and vulnerability. Crop Sci..

[8] Pessarakli M. (2007). Handbook of Turfgrass Management and Physiology.

[9] Turgeon A. (2008). Turfgrass Management.

[10] Zheng Y., Li F., Hao L., Yu J., Guo L., Zhou H., Ma C., Zhang X., Xu M. (2019). Elevated CO_2_ concentration induces photosynthetic down-regulation with changes in leaf structure, non-structural carbohydrated and nitrogen content of soybean. BMC Plant Biol..

[11] Pedersen J., Santos F.D., Vuuren D.V., Gupta J., Swart R. (2021). An assessment of the performance of scenarios against historical global emissions for IPCC reports. Global Environ. Chang..

[12] Huang B., Xu Y. (2015). Cellular and molecular mechanisms for elevated CO_2_-regulation of plant growth and stress adaptation. Crop Sci..

[13] Yu J.J., Chen L.H., Xu M., Huang B.R. (2012). Effects of elevated CO_2_ on physiological responses of tall fescue to elevated temperature, drought stress, and the combined stresses. Crop Sci..

[14] Yu J.J., Yang Z.M., Jespersen D., Huang B.R. (2014). Photosynthesis and protein metabolism associated with elevated CO_2_-mitigation of heat stress damages in tall fescue. Environ. Exp. Bot..

[15] Yu J., Fan N., Li R., Zhuang L., Xu Q., Huang B. (2019). Proteomic profiling for metabolic pathways involved in interactive effects of elevated carbon dioxide and nitrogen on leaf growth in a perennial grass species. J. Proteome Res..

[16] Chen Y.J., Yu J.J., Huang B.R. (2015). Effects of elevated CO_2_ concentration on water relations and photosynthetic responses to drought stress and recovery during rewatering in tall fescue. J. Am. Soc. Hortic. Sci..

[17] Song Y.L., Yu J.J., Huang B. (2014). Elevated CO_2_-mitigation of high temperature stress associated with maintenance of positive carbon balance and carbohydrate accumulation in kentucky bluegrass. PLoS ONE.

[18] Zhuang L., Yang Z., Fan N., Yu J., Huang B. (2019). Metabolomic changes associated with elevated CO_2_-regulation of salt tolerance in Kentucky bluegrass. Environ. Exp. Bot..

[19] Yu J., Li R., Fan N., Yang Z., Huang B. (2017). Metabolic pathways involved in carbon dioxide enhanced heat tolerance in bermudagrass. Front. Plant Sci..

[20] Yu J., Sun L., Fan N., Yang Z., Huang B. (2015). Physiological factors involved in positive effects of elevated carbon dioxide concentration on bermudagrass tolerance to salinity stress. Environ. Exp. Bot..

[21] Burgess P., Huang B. (2014). Growth and physiological responses of creeping bentgrass (*Agrostis stolonifera*) to elevated carbon dioxide concentrations. Hortic. Res..

[22] Kinmonth-Schultz H., Kim S.H. (2011). Carbon gain, allocation, and storage in rhizomes in response to elevated CO_2_ and fertilization in an invasive perennial C_3_ grass, *Phalaris arundinacea*. Func. Plant Biol..

[23] Kondhare K.R., Patil A.B., Giri A.P. (2021). Auxin: An emerging regulator of tuber and storage root development. Plant Sci..

[24] Mu X., Chen Q., Wu X., Chen F., Yuan L., Mi G. (2018). Gibberellins synthesis is involved in the reduction of cell flux and elemental growth rate in maize leaf under low nitrogen supply. Environ. Exp. Bot..

[25] Liu D., Xu M., Hu Y., Wang R., Tong J., Xiao L. (2019). Dynamic changes of key plant hormones during potato tuber development. Mol. Plant Breed..

[26] Wu W., Kang D., Kang X., Wei H. (2021). The diverse roles of cytokinins in regulating leaf development. Hortic. Res..

[27] Burgess P., Chapman C., Zhang X., Huang B. (2019). Stimulation of growth and alteration of hormones by elevated carbon dioxide for creeping bentgrass exposed to drought. Crop Sci..

[28] Elgersma A., Li F. (1997). Effects of cultivar and cutting frequency on dynamics of stolon growth and leaf appearance in white clover in mixed swards. Grass Forage Sci..

[29] Tworkoski T.J., Benassi T.E., Takeda F. (2001). The effect of nitrogen on stolon and ramet growth in four genotypes of *Fragaria chiloensis* L. Sci. Hortic..

[30] O’Neal S.W., Prince J.S. (1988). Seasonal effects of light, temperature, nutrient concentration and salinity on the physiology and growth of *Caulerpa paspaloides* (Chlorophyceae). Marine Biol..

[31] Dong B.C., Yu G.L., Wei G., Zhang M.X., Dong M., Yu F.H. (2010). How internode length, position and presence of leaves affect survival and growth of *Alternanthera philoxeroides* after fragmentation?. Ecol. Evol..

[32] Dong B., Liu R., Zhang Q., LI H., Zhang M., Lei G., Yu F. (2011). Burial depth and stolon internode length independently affect survival of small clonal fragments. PLoS ONE.

[33] Chapman C., Burgess P., Huang B. (2021). Effects of elevated carbon dioxide on drought tolerance and post-drought recovery involving rhizome growth in kentucky bluegrass (*Poa pratensis* L.). Crop Sci..

[34] Lawson A.R., Kelly K.B., Sale P. (2000). Defoliation frequency and cultivar effects on the storage and utilisation of stolon and root reserves in white clover. Aust. J. Agr. Res..

[35] Goulas E., Le Dily F., Teissedre L., Corbel G., Robin C., Christophe R., Ourry A. (2001). Vegetative storage proteins in white clover (*Trifolium repens* L.): Quantitative and qualitative features. Ann. Bot..

[36] Huang Q., Shen Y., Li X., Zhang G., Huang D., Fan Z. (2014). Regeneration capacity of the small clonal fragments of the invasive *Mikania micrantha* H.B.K.: Effects of the stolon thickness, internode length and presence of leaves. Weed Biol. Manag..

[37] Urbonaviciute A., Samuoliene G., Sakalauskaite J., Duchovskis P., Brazaityte A., Siksnianiene J.B., Ulinskaite R., Sabajeviene G., Baranauskis K. (2006). The effect of elevated CO_2_ concentrations on leaf carbohydrate, chlorophyll contents and photosynthesis in radish. Pol. J. Environ. Stud..

[38] Pérez-López U., Robredo A., Lacuesta M., Mena-Petite A., Muñoz-Rueda A. (2012). Elevated CO_2_ reduces stomatal and metabolic limitations on photosynthesis caused by salinity in *Hordeum vulgare*. Photosynth. Res..

[39] Faria T., Wilkins D., Besford R.T., Vaz M., Pereira J.S., Chaves M.M. (1996). Growth at elevated CO_2_ leads to down-regulation of photosynthesis and altered response to high temperature in *Quercus suber* L. seedlings. J. Exp. Bot..

[40] Jach M.E., Ceulemans R. (1999). Effects of elevated atmospheric CO_2_ on phenology, growth and crown structure of Scots pine (*Pinus sylvestris*) seedlings after two years of exposure in the field. Tree Physiol..

[41] Li X., Shen Y., Huang Q., Fan Z., Huang D. (2013). Regeneration capacity of small clonal fragments of the invasive *Mikania micrantha* H.B.K.: Effects of burial depth and stolon internode length. PLoS ONE.

[42] Zhou Y., Lambrides C., Fukai S. (2015). Associations between drought resistance, regrowth and quality in a perennial C_4_ grass. Eur. J. Agron..

[43] He L., Xiao X., Zhang X., Jin Y., Pu Z., Lei N., He X., Chen J. (2021). Clonal fragments of stoloniferous invasive plants benefit more from stolon storage than their congeneric native species. Flora.

[44] Fry J.D., Lang N.S., Clifton R., Maier F.P. (1993). Freezing tolerance and carbohydrate content of low-temperature-acclimated and nonacclimated centipedegrass. Crop Sci..

[45] Patton A.J., Volenec J.J., Reicher Z.J. (2007). Stolon growth and dry matter partitioning explain differences in zoysiagrass establishment rates. Crop Sci..

[46] Chapman C., Burgess P., Huang B. (2021). Responses to elevated carbon dioxide for postdrought recovery of turfgrass species differing in growth characteristics. Crop Sci..

[47] Burgess P., Huang B. (2014). Root protein metabolism in association with improved root growth and drought tolerance by elevated carbon dioxide in creeping bentgrass. Field Crop Res..

[48] Li T., Di Z., Han X., Yang X. (2012). Elevated CO_2_ improves root growth and cadmium accumulation in the hyperaccumulator *Sedum alfredii*. Plant Soil.

[49] Hiltpold I., Moore B., Johnson S. (2020). Elevated atmospheric carbon dioxide concentrations alter root morphology and reduce the effectiveness of entomopathogenic nematodes. Plant Soil.

[50] Da Cruz G.S., Audus L.J. (1978). Studies of hormone-directed transport in decapitated stolons of *Saxifraga sarmentosa*. Ann. Bot..

[51] Braun J., Kender W. (1985). Correlative bud inhibition and growth habit of the strawberry as influenced by application of gibberellic acid, cytokinin, and chilling during short daylength. J. Am. Soc. Hortic. Sci..

[52] Tyagi S., Kumar S. (2016). Exogenous supply of IAA, GA and cytokinin to salinity stressed seeds of chickpea improve the seed germination and seedling growth. Int. J. Plant Sci..

[53] Kotov A.A., Kotova L.M., Romanov G.A. (2021). Signaling network regulating plant branching: Recent advances and new challenges. Plant Sci..

[54] Adams R.E., Kerber K., Pfister E.W., Weiler E.W., Karssen C.M., van Loon L.C., Vreugdenhil D. (1992). Studies on the action of the new growth retardant CGA 163′935 (Cimectacarb). Progress in Plant Growth Regulation.

[55] Reicher Z.J., Dernoeden P.H., Richmond D.S., Stier J.C., Horgan B.P., Bonos S.A. (2013). Insecticides, fungicides, herbicides, and growth regulators used in turfgrass systems. Turfgrass: Biology, Use, and Management.

[56] McCann S.E., Huang B. (2007). Effects of trinexapac-ethyl foliar application on creeping bentgrass responses to combined drought and heat stress. Crop Sci..

[57] McCann S.E., Huang B. (2008). Drought responses of Kentucky bluegrass and creeping bentgrass as affected by abscisic acid and trinexapac-ethyl. J. Am. Soc. Hortic. Sci..

[58] Hoagland D.R., Arnon D.I. (1950). The Water-Culture Method for Growing Plans without Soil.

[59] Liu N., Shen Y., Huang B. (2015). Osmoregulants involved in osmotic adjustment for differential drought tolerance in different bentgrass genotypes. J. Am. Soc. Hortic. Sci..

[60] Pan X., Welti R., Wang X. (2010). Quantitative analysis of major plant hormones in crude plant extracts by high-performance liquid chromatography-mass spectrometry. Nat. Protoc..

